# Crossed Entorhino-Dentate Projections Form and Terminate With Correct Layer-Specificity in Organotypic Slice Cultures of the Mouse Hippocampus

**DOI:** 10.3389/fnana.2021.637036

**Published:** 2021-02-11

**Authors:** Lars Hildebrandt-Einfeldt, Kenrick Yap, Mandy H. Paul, Carolin Stoffer, Nadine Zahn, Alexander Drakew, Maximilian Lenz, Andreas Vlachos, Thomas Deller

**Affiliations:** ^1^Institute of Clinical Neuroanatomy, Dr. Senckenberg Anatomy, Neuroscience Center, Goethe-University Frankfurt, Frankfurt, Germany; ^2^Department of Neuroanatomy, Institute of Anatomy and Cell Biology, Faculty of Medicine, University of Freiburg, Freiburg, Germany; ^3^Center Brain Links Brain Tools, University of Freiburg, Freiburg, Germany; ^4^Center for Basics in Neuro Modulation (NeuroModulBasics), Faculty of Medicine, University of Freiburg, Freiburg, Germany

**Keywords:** calretinin, viral tracing, perforant path transection, species differences, layer-specificity, dentate gyrus

## Abstract

The entorhino-dentate projection, i.e., the perforant pathway, terminates in a highly ordered and laminated fashion in the rodent dentate gyrus (DG): fibers arising from the medial entorhinal cortex (MEC) terminate in the middle molecular layer, whereas fibers arising from the lateral entorhinal cortex (LEC) terminate in the outer molecular layer of the DG. In rats and rabbits, a crossed entorhino-dentate projection exists, which originates from the entorhinal cortex (EC) and terminates in the contralateral DG. In contrast, in mice, such a crossed projection is reportedly absent. Using single and double mouse organotypic entorhino-hippocampal slice cultures, we studied the ipsi- and crossed entorhino-dentate projections. Viral tracing revealed that entorhino-dentate projections terminate with a high degree of lamina-specificity in single as well as in double cultures. Furthermore, in double cultures, entorhinal axons arising from one slice freely intermingled with entorhinal axons originating from the other slice. In single as well as in double cultures, entorhinal axons exhibited a correct topographical projection to the DG: medial entorhinal axons terminated in the middle and lateral entorhinal axons terminated in the outer molecular layer. Finally, entorhinal neurons were virally transduced with Channelrhodopsin2-YFP and stimulated with light, revealing functional connections between the EC and dentate granule cells. We conclude from our findings that entorhino-dentate projections form bilaterally in the mouse hippocampus *in vitro* and that the mouse DG provides a permissive environment for crossed entorhinal fibers.

## Introduction

Entorhino-dentate axons relay cortical information from the entorhinal cortex (EC) to the dentate gyrus (DG; e.g., Van Hoesen et al., 1972; Wilson and Steward, 1978; Jones, 1993; Förster et al., 2006; Nilssen et al., 2019; Lee et al., 2020). Anatomically, the entorhino-dentate connections are subdivided into two major projections: one projection arises from layer II neurons in the medial entorhinal cortex (MEC) and its axons terminate in the middle molecular layer of the DG. The other projection arises from layer II neurons in the lateral entorhinal cortex (LEC) and its axons terminate more superficially in the outer molecular layer of the dentate. Using a series of micro-DiI injections, Tamamaki (1997) demonstrated that the laminar termination of the two projections within the DG is very precise and that the medial to the lateral organization of the EC is converted into a vertical stack of axonal layers reaching from the deep to the superficial parts of the molecular layer of the DG (Tamamaki, 1997). Thus, a high degree of topographical organization exists between the EC and the DG.

In rats (Steward et al., 1974; Zimmer and Hjorth-Simonsen, 1975; Steward, 1976; Deller et al., 1996) and rabbits (Hjorth-Simonsen and Zimmer, 1975) a crossed projection from the EC to the DG also exists. In naïve rats, this projection is much weaker than the non-crossed (“ipsilateral” projection). However, following ipsilateral entorhinal denervation, the remaining crossed entorhino-dentate axons branch and sprout new collaterals (Deller et al., 1996). The axonal and synaptic reorganization occurring after denervation results in a 100-fold increase in crossed entorhino-dentate synapses (Cotman et al., 1977; Steward et al., 1988). The expanded projection also becomes functionally relevant and exhibits functional properties, which are similar to the ipsilateral, i.e., lost, entorhinal projection (Steward et al., 1973, 1974; Steward, 1976; Reeves and Steward, 1986). Thus, the crossed entorhino-dentate projection is an excellent example of the ability of the brain to remodel its connections and to achieve a certain degree of functional recovery following lesion using network reorganization strategies.

In contrast to rats, a crossed EC-DG projection could not be demonstrated in mice (van Groen et al., 2002, 2003) and its absence has been regarded as one of several anatomical differences between the two species (van Groen et al., 2002; Deller et al., 2007). Why mice do not form a crossed EC-DG projection is presently unclear. Repellent factors within the contralateral DG, for example within the extracellular matrix (Deller et al., 2000), absence of crossed pioneer axons arising from Cajal-Retzius cells (del Rio et al., 1997; Ceranik et al., 1999), or negative interactions with axons (Kolodkin, 1996; Skutella and Nitsch, 2001) of the contralateral EC projection are possibilities. To test the hypothesis that signals within the DG could prevent the ingrowth of EC fibers from the contralateral EC, we co-cultivated two organotypic slice cultures consisting of mouse EC-DG each and allowed these cultures to mature. As has been shown previously, such cultures form extensive commissural/associational projections (Frotscher and Heimrich, 1993; Frotscher et al., 1995; Del Turco et al., 2019), in effect forming a bilateral hippocampal formation *in vitro*. We, therefore, predicted that in mouse EC-DG double cultures, axons from the contralateral EC should not be able to enter the DG, if the DG contains repellent signals. However, in the absence of such signals, crossed EC fibers should enter the DG and interact with axons from the ipsilateral EC, raising the question, whether these crossing fibers terminate with the same layer specificity and intermingle with EC axons from the ipsilateral side.

## Materials and Methods

### Animals

Wild-type mice (C57Bl6/J) were used in this study. All animal experiments were performed following the German animal welfare law and had been declared to the Animal Welfare Officer of the Faculty (Wa-2014-35). Mice were bred and housed at the animal facility of the Goethe-University Hospital Frankfurt and were maintained on a 12 h light/dark cycle with food and water available ad libitum. Every effort was made to minimize the distress and pain of animals.

### Organotypic Slice Cultures

Organotypic entorhino-hippocampal slice cultures were prepared at postnatal day 4–5 from C57Bl6/J wildtype mouse brain of either sex based on previously published protocols (Del Turco and Deller, 2007). To prepare single cultures, animals were decapitated and slices containing both, EC and hippocampus, were dissected from 300 μm thick vibratome sections (VT1200S, Leica). Preparation medium for brain dissection contained Minimal essential medium (MEM, Gibco) including 2 mM glutamax (Gibco), 25 mM HEPES (Gibco), 0.45% (w/v) glucose (Sigma–Aldrich), 100 U/ml penicillin (Sigma–Aldrich) and 0.1 mg/ml streptomycin (Sigma–Aldrich, pH 7.3–7.4). Slice cultures were maintained on porous-membrane filter inserts (Millicell-CM; Millipore) and incubated in a humidified atmosphere with 5% CO_2_ at 35°C. For double EC-DG slice cultures, two entorhino-hippocampal slices were positioned in contact with each other on the membrane filter insert. The medial aspects of the two slices touched and the axes of the two cultures were oriented in parallel, i.e., the DG of one culture was placed next to the DG of the second culture. Cultivation medium contained 42% (v/v) MEM, 25% (v/v) basal medium eagle (BME, Gibco), 25% (v/v) heat-inactivated normal horse serum (NHS, GibcoBRL), 25 mM HEPES (Gibco), 0.15% (w/v) bicarbonate (Invitrogen), 0.675% (w/v) glucose (Sigma–Aldrich), 0.1 mg/ml streptomycin (Sigma–Aldrich), 100 U/ml penicillin (Sigma–Aldrich) and 2 mM glutamax (Gibco). The pH was adjusted to 7.3 and the medium was changed every 2–3 days.

### Perforant Path Lesion *In vitro*

Entorhino-hippocampal cultures were allowed to mature until 28 days *in vitro* (DIV). As described elsewhere (Del Turco and Deller, 2007; Del Turco et al., 2019), entorhino-hippocampal projections were completely transected utilizing a sterile scalpel blade (first lesion: 28 DIV; second lesion: 35 DIV; end of incubation and fixation: 42 DIV).

### Adeno-Associated Virus Production

Pseudotyped adeno-associated viral (AAV) particles were generated using a helper virus-free packaging method. HEK293T cells were simultaneously transfected with pDP1rs (Plasmid Factory), pDG (Plasmid Factory), and either an AAV2-hSyn1-GFP or AAV2-hSyn1-tdTomato vector plasmid (Shevtsova et al., 2005; 12:8:5) using calcium phosphate precipitation (protocol adapted from Grimm et al., 1998; Grimm, 2002). Cells were collected 48 h after transfection, washed twice with Phosphate Buffered Saline (PBS), centrifuged at 1,200 rpm for 10 min, and re-suspended in PBS. Recombinant viral particles within the cells were released by four freeze- and thaw cycles and the supernatant was centrifuged at 10,000 rpm for 10 min to remove cell debris. In a final step, the supernatant was collected, aliquoted, and stored at −80°C.

### Local Injections of Adeno-Associated Viruses into the Entorhinal Cortex

To transduce and thus label neurons in the EC, slice cultures were injected (DIV 3–5) with AAV2-hSyn1-GFP or AAV2-hSyn1-tdTomato virus. The EC was visually identified in slices based on its location and morphology. Injections into the putative MEC (medial part of the EC close to the angular bundle) and the putative LEC (lateral part of the EC) were performed under visual control. Local injections were performed using an injection pipette pulled from thin-walled borosilicate capillaries (Harvard Apparatus, 30-0066). A head stage with an HL-U holder (Axon Instruments) was used to hold the pipettes. Pipettes were positioned over the EC using a micro-manipulator (Luigs and Neumann). Approximately 0.05–0.1 μl of the virus-containing solution was pressure-injected into the region of interest in the EC. To visualize the slice culture and to position the pipette, an upright microscope (Nikon FN1) containing a 10× water immersion objective lens (Nikon Plan Fluor, NA 0.30) was used.

### Immunofluorescence

Slice cultures were fixed in a solution of 4% (w/v) paraformaldehyde (PFA) in phosphate-buffered saline (PBS; 0.1 M, pH 7.4) and 4% (w/v) sucrose for 1 h, followed by 2% PFA and 30% sucrose in PBS overnight. After several washes with 0.1 M PBS (pH 7.4), cultures were re-sliced into 30 μm sections on a cryostat (CM3050S, Leica). Free-floating sections were washed several times in 0.1 M PBS. The sections were incubated thereafter for 1 h with 10% (v/v) normal goat serum (NGS) in 0.5% (v/v) Triton X-100 containing PBS to reduce unspecific staining. Some slices were also incubated for 48 h at 4°C with the primary antibody (rabbit anti-Calretinin, Swant, 1:500) in PBS with 5% NGS and 0.1% Triton X-100. The sections were washed several times and incubated for 4 h with appropriate Alexa488 or Alexa633-labeled secondary antibodies (Invitrogen; 1:1,000 or 1:500) in PBS with 5% NGS and 0.1% Triton X-100). TO-PRO (Invitrogen) nuclear stain was used to visualize cytoarchitecture (1:5,000; in PBS for 10 min). The sections were washed, transferred on glass slides, and mounted for visualization with an anti-fading mounting medium (Dako, Agilent Technologies).

### Confocal Microscopy of Fixed Slice Cultures

Images were obtained at a resolution of 1,024 × 1,024 pixels using a confocal laser scanning microscope (Nikon Eclipse 80i; 488 nm and 561 nm excitation lasers; EZ-C1 3.60 software) equipped with a 4× objective lens [numeric aperture (NA) 0.2, Nikon], a 10× objective lens (NA 0.3, Nikon), and a 20× objective lens (NA 0.75, Nikon). Detector gain and amplifier were set to obtain pixel intensities within a linear range.

### Time-Lapse Imaging

Live imaging of slice cultures was performed as previously described, with modifications (Del Turco et al., 2019). The membrane insert with the cultures was placed into a 30 mm petri dish that contained a warm (37°C) imaging medium, which consisted of NaCl 129 mM, KCl 4 mM, MgCl_2_ 1 mM, CaCl_2_ 2 mM, glucose 4.2 mM, HEPES 10 mM, Trolox 0.1 mM, streptomycin 0.1 mg/ml, and penicillin 100 U/ml; pH 7.4. The osmolarity of the imaging medium was adjusted with sucrose to the osmolarity of the incubation medium. Imaging was done with an upright confocal microscope (ZEISS, LSM Pascal; 488 nm excitation laser) equipped with a temperature-regulated stage (37°C), using a 10× water immersion objective lens (NA 0.3; Zeiss) to visualize slice cultures and to identify AAV-labeled EC cells (layer II/III) and their projections to the DG. Image stacks (approximately 20 images per stack; *z*-axis interval between consecutive frames: 5 μm) of DG regions were obtained at a resolution of 1,024 × 1,024 pixels (0.7× zoom, 1.77 × 1.77 μm pixel size). Slice cultures were imaged on 18, 21, 28, 35, and 42 DIV for less than 10 min per culture to keep exposure time and phototoxic damage minimal.

### Digital Illustrations

Confocal image stacks were stored as lsm-files (ZEISS) and ics/ids (Image Cytometry Standard; Nikon) file format. Images were edited with Fiji (Image Processing and Analysis in Java, version 1.48s; Schindelin et al., 2012). Figures were prepared with commercially available graphics software (Photoshop, Adobe Inc., San Jose, CA, USA). The contrast and brightness were adjusted. No additional image alteration was performed.

### Photostimulation and Electrophysiological Recordings

The medial EC of entorhino-hippocampal slice cultures was injected with pAAV-CaMKIIa-hChR2(H134R)-EYFP (gift from Karl Deisseroth; Addgene plasmid #26969^1^; RRID: Addgene_26969; (Lee et al., 2010) on 2–5 DIV using an Eppendorf Transjector 5246. The virus-containing solution was diluted to 5*10^12^ gene copies (GC) in PBS. Three injections were made per culture (~100 mbar; 7 s), with ~150 μm distance between single injection sites.

Patch-clamp recordings were conducted ~4 weeks after viral transduction. Entorhino-hippocampal slice cultures were continuously perfused with artificial cerebrospinal fluid (ACSF; 126 mM NaCl, 2.5 mM KCl, 2 mM CaCl_2_, 2 mM MgCl_2_, 1.25 mM NaH_2_PO_4_, 26 mM NaHCO_3_, 10 mM glucose) and oxygenated with 95% O_2_/5% CO_2_ in a temperature-controlled recording chamber (Luigs and Neumann) at 35°C. Patch pipettes (3–5 MΩ) were pulled from borosilicate glass (GC150TF, Harvard Apparatus) and filled with the internal solution (126 mM K-Gluconate, 4 mM KCl, 0.3 mM Na_2_-GTP, 4 mM Mg-ATP, 10 mM PO-Creatine, 10 mM HEPES; pH 7.25). Whole-cell current-clamp recordings were acquired from hippocampal granule cells, which were visualized by infrared differential interference contrast microscopy (Zeiss Axioskop 2FS, 40× water immersion objective lens, NA 0.75). For optical stimulation, a 447.5 nm LED (SP-03-V4, Luxeon Star), controlled by a self-made Rasberry-Pi-driven controller unit, was positioned 20 mm away from the culture. ChR2-eYFP expressing neurons were stimulated with single 10 ms light pulses with a light intensity of 7.5 mW/mm^2^. Data were acquired using a MultiClamp 700B amplifier (Axon Instruments) and digitized at a sampling rate of 10 kHz using the DigiData 1440A interface (Axon Instruments).

## Results

### Entorhinal Axons Terminate With Correct Laminar-Specificity in the DG of Single Organotypic EC-DG Slice Cultures

**Figure 1:**
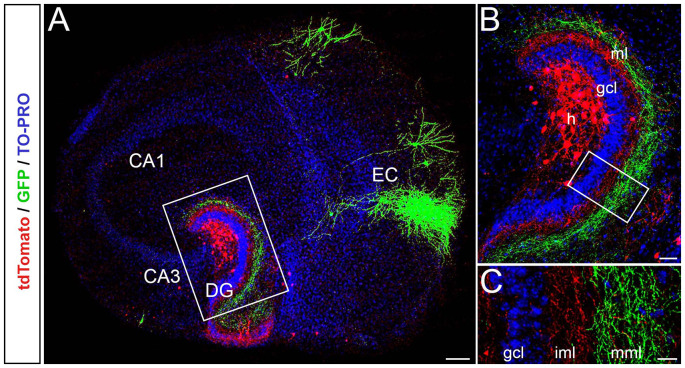
Entorhinal axons terminate with correct laminar-specificity in the dentate gyrus (DG) of single organotypic EC-DG slice cultures obtained from mouse brain. **(A)** Viral tracings of entorhinal cortical and associational fibers in the DG (red, AAV2-hSyn-tdTomato; green, AAV2-hSyn-GFP; TO-PRO Iodide nuclear stain, blue) reveal the entorhino-dentate (green) and the associational projections (red) in single organotypic slice cultures. EC, entorhinal cortex; CA3 and CA1; hippocampal subfields CA3 and CA1. Scale bar: 200 μm. **(B)** Higher magnification of the rectangle in panel **(A)**. Entorhino-dentate axons and associational axons, i.e., putative mossy cell axons, are visualized. Note the layer-specific termination of entorhinal axons in the outer parts of the molecular layer and associational axons in the inner parts of the molecular layer. GCL, granule cell layer; h, hilus. Scale bar: 50 μm. **(C)** Higher magnification of the rectangle in panel **(B)** showing the lamina-specific termination of the two fiber systems. Some red axons, most likely arising from interneurons in the hilus, are seen within the “entorhinal zone” of the DG. Scale bar: 25 μm.

To study the laminar termination pattern of EC-DG axons in single organotypic EC-DG slice cultures, microinjections of GFP-AAV into the EC, and microinjections of tdTomato-AAV into the hilus of the DG were performed (Figure 1A). This allowed us to visualize single entorhinal and associational axons in the molecular layer of the DG and to study their termination zones. The two fiber plexus terminated in a lamina-specific and complementary fashion, i.e., entorhinal axons in the middle and outer third of the molecular layer and associational axons in the inner molecular layer (Figure 1B). Some associational axons were also seen in the “entorhinal zone” of the DG (Figure 1C). These axons most likely arise from “non-mossy cells” in this region, such as neuropeptide Y (NPY) or Somatostatin (SOM)-containing interneurons (Deller and Leranth, 1990; Leranth et al., 1990; Freund and Buzsàki, 1996).

**Figure 2:**
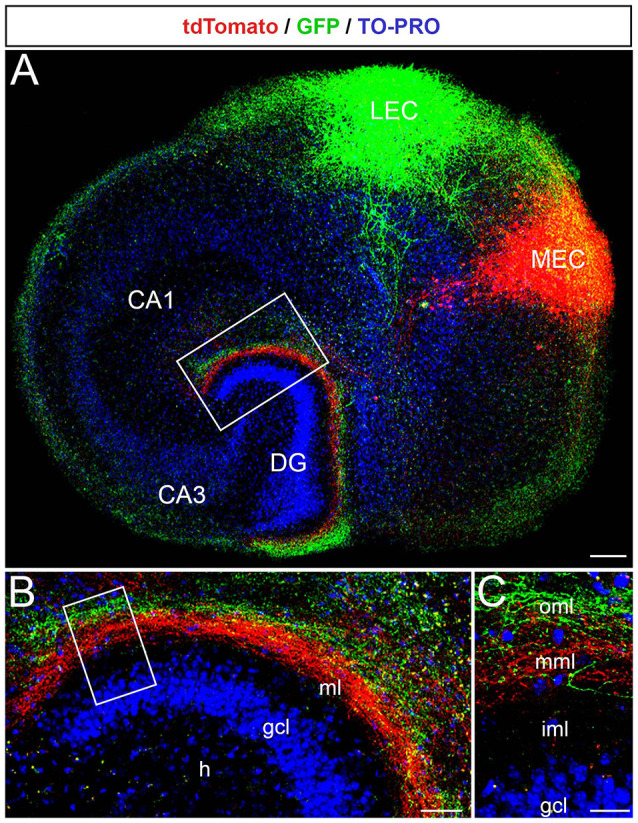
The laminar-termination of axons from the medial entorhinal cortex (MEC) and lateral entorhinal cortex (LEC) is preserved in single organotypic EC-DG slice cultures. **(A)** Viral tracings of entorhinal fibers from the MEC (red, AAV2-hSyn-tdTomato) and the LEC (green, AAV2-hSyn-GFP) revealed the laminar termination pattern of fibers from the two entorhinal areas in the DG. Cell layers in the culture were visualized by TO-PRO Iodide nuclear stain (blue). CA3, CA1: hippocampal subfields. Scale bar: 200 μm. **(B)** The rectangle in panel **(A)** shown at higher magnification. MEC axons terminate in deeper parts of the molecular layer (ml), whereas LEC axons terminate more superficially. GCL, granule cell layer; H, hilus. Scale bar: 50 μm. **(C)** The rectangle in panel **(B)** is shown at higher magnification. iml, inner molecular layer; mml, middle molecular layer; oml, outer molecular layer. Scale bar: 25 μm.

Next, we microinjected the two AAVs into the putative MEC and LEC to study their projections (Figure 2A). As described for the *in vivo* situation, axons arising from neurons in medial parts of the EC terminated in the middle molecular layer, whereas axons arising from neurons in lateral parts of the EC terminated in the outer molecular layer of the DG (Figures 2A,B). The two fiber plexus did not overlap (Figure 2C). Thus, the EC-DG projection maintains its topographical organization in organotypic EC-DG cultures from the mouse brain.

### Crossed Entorhino-Dentate Projections Form Between Two Adjacent Organotypic EC-DG Slice Cultures

**Figure 3:**
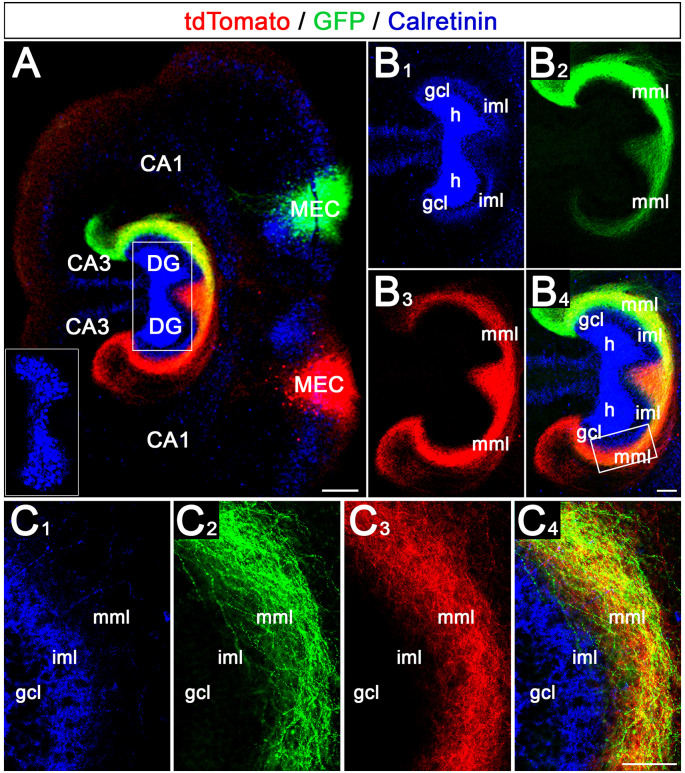
Crossed entorhino-dentate projections form between two adjacent organotypic EC-DG slice cultures. **(A)** Using viral tracing of the entorhino-hippocampal projection in double cultures, the relationship of axons arising from the two medial entorhinal cortices (MEC) were studied. One MEC projection was labeled green (AAV2-hSyn-GFP) whereas the second MEC projection was labeled red (AAV2-hSyn-tdTomato). Calretinin-immunostaining was employed to visualize the mossy cells and their axons in the inner molecular layer (iml; blue). Note that the blue channel was deliberately overexposed to visualize the calretinin-positive axons in the iml. The inset shows the distribution of the hilar mossy cells (shorter exposure time). CA3, CA1: hippocampal subfields CA3, CA1; DG, dentate gyrus. Scale bar: 200 μm. **(B1–B4)** Termination patterns of Calretinin- **(B1)**, GFP- **(B2)**, and tdTomato- **(B3)** labeled axons. An overlay is shown in panel **(B4)**. Note that the axon plexus in the middle molecular layer (mml) is denser on the side of the injection (e.g., **B2**,**B3**). On the ipsilateral side, the axon plexus covers the entire mml and extends into stratum lacunosum-moleculare of area CA3. On the contralateral side, the crossed axon plexus is densest in the proximal mml and gradually becomes weaker distally **(B2,B3)**. gcl, granule cell layer. Scale bar: 100 μm. **(C1–C4)** Region of (**B1–B4**) indicated by the rectangle in panel **(B4)** shown at higher magnification **(C1–C4)**. Fibers from both MECs intermingle within the mml and form a border towards the calretinin-plexus in the iml. Scale bar: 50 μm.

In a second step, we tested whether a crossed projection forms between the EC of one EC-DG slice culture and the DG of a second EC-DG slice culture positioned adjacent to it. A GFP-AAV was injected into the MEC of one culture and a tdTomato-AAV was injected into the MEC of the other culture (Figure 3A). Also, fixed double cultures were stained for calretinin to label the associational/commissural mossy cell axons in the inner molecular layer (Figure 3A). This made it possible to address the following questions: (i) does a crossed projection form? (ii) if yes, do entorhinal axons from both sides intermingle? and (iii) do they respect the border to the inner molecular layer?

Our experiments revealed that crossed entorhino-dentate projections form bilaterally *in vitro* (Figure 3). Of note, on both sides, the ipsilateral projection, i.e., the EC projection to the ipsilateral DG, was stronger and more pronounced than the crossed projection. The ipsilateral projection covered the entire “entorhinal zone” of the molecular layer and area CA3, whereas the crossed projections became gradually weaker and faded away towards stratum lacunosum-moleculare of CA3. Within the middle molecular layer of both DGs entorhinal axons from both sides readily intermingled and terminated within the same layer (Figure 3C). Furthermore, both projections respected the border to the inner molecular layer, which we visualized using calretinin-immunolabeling of mossy cell axons. In sum, our data show that *in vitro* a crossed projection forms bilaterally, crossed fibers terminate together with ipsilateral fibers within their normal territory, and both systems respect the border towards the inner molecular layer.

### Crossed Entorhino-Dentate Axons Arising From Medial and Lateral Parts of the EC Terminate With Correct Topographical Specificity in the Contralateral DG

**Figure 4:**
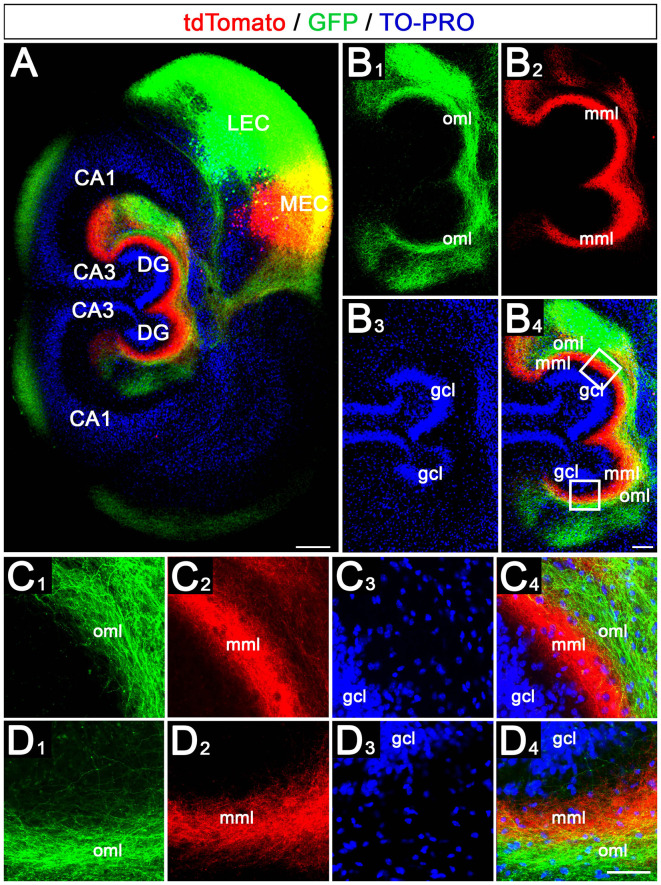
Crossed entorhino-dentate axons arising from MEC and LEC terminate with correct topographical specificity in the contralateral DG. **(A)** Viral tracing was again employed to study the ipsi- and contralateral termination of LEC (green, AAV2-hSyn1-GFP) and MEC (red, AAV2-hSyn1-tdTomato) axons in EC-DG double cultures. Injections were placed into one EC only. TO-PRO was used to visualize hippocampal cell layers. Scale bar: 200 μm. **(B1–B4)** Higher magnification of the DG shown in panel **(A)**. gcl, granule cell layer; mml, middle molecular layer; oml, outer molecular layer. Scale bar: 100 μm. **(C1–C4)** Higher magnification of the upper boxed area in panel **(B4)**. Axons from the LEC terminate in the oml, whereas axons arising from the MEC terminate in the mml of the DG. Scale bar: 50 μm. **(D1–D4)** Higher magnification of the lower boxed area in panel **(B4)**. Axons from the LEC and MEC terminate in the oml and mml, respectively. Scale bar: 50 μm.

We then wondered whether the crossed projection from the EC to the DG maintains the medial to the lateral topographical organization of the EC projections. For this, we injected the AAVs into the putative MEC and LEC on one side and studied the termination pattern of these projections in the ipsi- and contralateral DG (Figure 4).

This revealed on the side of the injection the same laminar-specific termination pattern as in single cultures, i.e., axons from medial EC neurons terminated in the middle molecular layer whereas axons from lateral EC neurons terminated in the outer molecular layer (Figures 4A–C). On the contralateral side, the crossed axons followed the same rules and terminated with the same high degree of lamina-specificity (Figures 4A,B,D): crossed medial EC axons terminated in the contralateral middle molecular layer and crossed lateral EC axons terminated superficially to this plexus in the outer molecular layer. Both projections remained distinct from each other.

### Sequential Transections of the EC-DG Projections Remove Entorhinal Afferents to the Ipsi- and Contralateral DG

**Figure 5:**
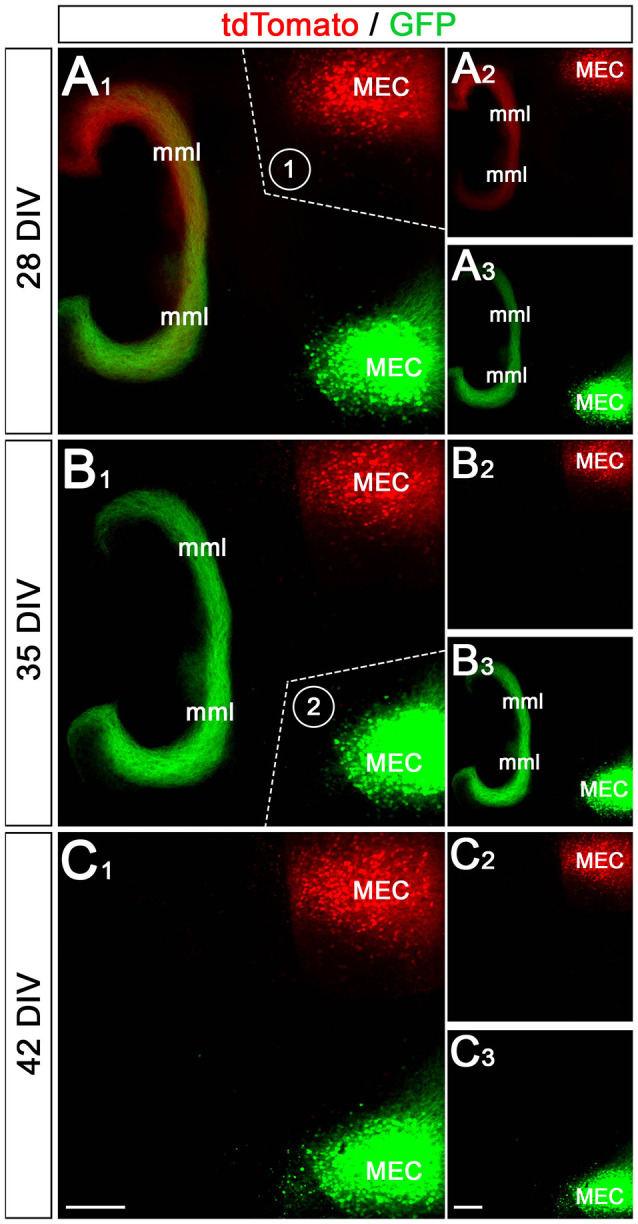
Sequential transection of EC projections removes entorhinal input to the ipsi- and contralateral DG. **(A1–A3)** Axons from the ipsi- and contralateral MEC are labeled with GFP (AAV2-hSyn-GFP) and tdTomato (AAV2-hSyn-tdTomato), respectively. At 28 days *in vitro* (DIV) both projections form a robust ipsi- and a weaker crossed entorhinal fiber plexus in the middle molecular layer (mml) of the two dentate gyri **(A2,A3)**. After imaging, the perforant path between the upper MEC (labeled in red) and the DG was cut (**A1**; dotted line, 1). **(B1–B3)** At 35 DIV the same cultures are re-imaged. Whereas the entorhinal axon plexus from the upper MEC is gone **(B2)**, the entorhinal plexus from the lower MEC (green) is still present **(B3)**. Following this imaging session, the second perforant path was cut (**B1**; dotted line, 2). **(C1–C3)** At 42 DIV both entorhinal projections are lost from the DG. Scale bars: **(C1**,**C3)**: 200 μm.

In the next step, we tested whether the EC-DG projections can also be selectively removed from the culture system (Figure 5). For this, we first transected the EC-DG projection on one side of the double-cultures on DIV 28 (Figure 5A) and, in a second step on DIV 35 the EC-DG projection on the other side of the double-culture (Figure 5B). The first transection removed all EC axons from the ipsi- or the contralateral hippocampus, belonging to the transected EC-DG projection. The axon plexus from the other side remained intact (Figure 5B). Following the second transection, all remaining EC axons were removed from the ipsi- or the contralateral hippocampus and on DIV 42 both DG were devoid of entorhinal axons (Figure 5C). This sequential lesioning model can be used in follow-up studies to analyze the sprouting of crossed entorhinal axons *in vitro*, as we previously did *in vivo* (Deller et al., 1996).

### Entorhino-Dentate Axons Form Functional Connections With Dentate Granule Cells

**Figure 6:**
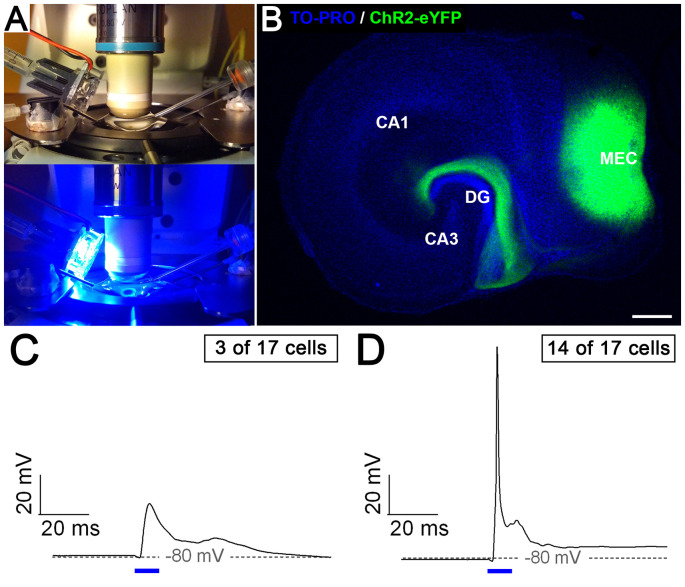
Entorhino-dentate axons form functional connections with dentate granule cells. **(A)** Organotypic slice cultures transduced with ChR2-eYFP were placed in a recording chamber and illuminated with blue light (447.5 nm). **(B)** Example of an entorhino-hippocampal slice culture after viral transduction of the medial entorhinal cortex (MEC) with ChR2-eYFP. Note the injection site in the MEC and the axon plexus in the DG. CA3, CA1, hippocampal subfields CA3 and CA1. Scale bar: 250 μm. Patch-clamp recordings of granule cells (*n* = 17 in six slice cultures) revealed either subthreshold **(C)** or suprathreshold **(D)** excitatory synaptic responses (EPSPs) to EC light stimulation (10 ms, indicated by blue bars).

Finally, we wondered whether the entorhino-dentate projections forming *in vitro* are, in fact, functional. We transduced entorhinal neurons with a ChR2-YFP construct using an adeno-associated vector system. This allowed us to stimulate the transduced EC neurons with light (Figure 6A) and to visualize their axonal projection to the DG (Figure 6B). Patch-clamp recordings from granule cells in the DG of such preparations showed synaptic responses to optical stimulation of the EC in all recorded granule cells (*n* = 17 in six slice cultures). Single Stimuli resulted in subthreshold (Figure 6C) as well as suprathreshold excitatory postsynaptic potentials (EPSPs; Figure 6D). Thus, the entorhino-dentate axons are functionally connected with dentate granule cells.

## Discussion

**Figure 7:**
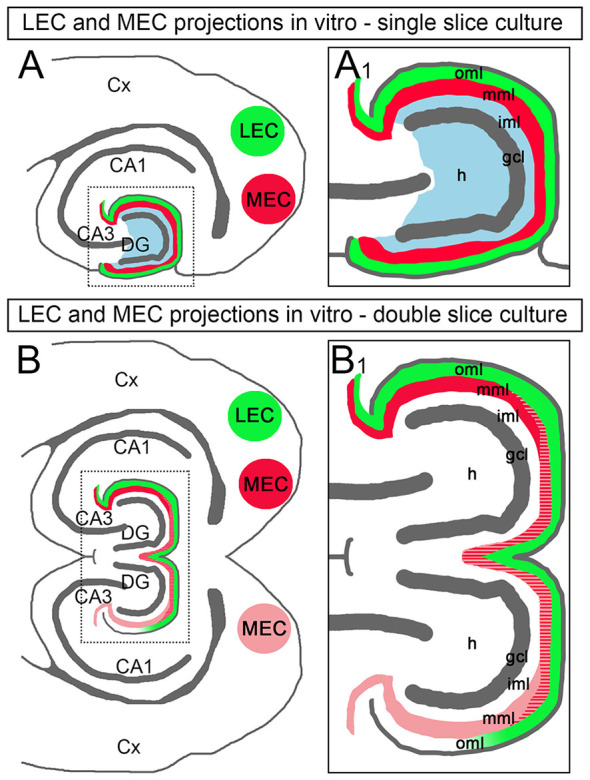
Schematic diagram illustrating the entorhinal projections in double EC-DG cultures of the mouse hippocampus. **(A)** In single EC-DG slice cultures, the topography of the projections to the DG is maintained. Axons arising from the LEC (green) terminate superficially in the outer molecular layer (oml), axons arising from the MEC terminate in the middle molecular layer (mml; red), and axons arising from the hilus (H) terminate in the inner molecular layer (iml; blue) of the DG. Cx, cortex; CA3, CA1: hippocampal subfields CA3 and CA1; gcl, granule cell layer. **(A1)** Boxed area in **(A)** shown at higher magnification. **(B)** In double EC-DG slice cultures, the topography of the EC projections is similarly maintained. Furthermore, EC-axons cross into the second culture, where they terminate in the correct layers and intermingle with locally arising EC axons. In many cases, the crossed projection was weaker than the ipsilateral projection and the axon plexus became weaker and eventually faded towards the distal oml and area CA3. **(B1)** Boxed area in **(B)** shown at higher magnification.

In the current study, we analyzed entorhino-dentate projections in single and double organotypic slice cultures of mouse EC and hippocampus. Our results can be summarized as follows: (1) the ipsilateral entorhino-dentate projection forms in organotypic slice cultures and maintains its layer-specific termination as well as its topographical organization; (2) *in vitro*, a crossed entorhino-dentate projection, which terminates with correct layer specificity and topography, is also present. This shows that the DG is permissive for ingrowing entorhinal fibers from the contralateral hemisphere; (3) ipsilateral and crossed entorhino-dentate fibers intermingle *in vitro*, demonstrating that ipsi- and crossed entorhinal fibers do not negatively interact with each other; and (4) after viral transduction of entorhinal neurons with ChR2, dentate granule cells could be activated with blue light, providing direct evidence for the functionality of the entorhino-dentate connection *in vitro*. In sum, we report that in mouse double entorhino-hippocampal slice cultures a bilateral entorhino-dentate system forms with normal specificity and topography (Figure 7).

The entorhino-dentate projection is one of the best-studied anatomical projections in the vertebrate brain. Although it has been revealed in all rodent species, subtle differences in the anatomical organization have been reported (Deller et al., 2007). Specifically, crossed entorhino-dentate fibers have been reported for rats (Steward et al., 1974) and rabbits (Hjorth-Simonsen and Zimmer, 1975) but not for mice (van Groen et al., 2002) using anterograde tracing techniques. We, therefore, wondered whether the mouse DG contains signals which prevent the ingrowth of fibers from the contralateral side. Our slice culture data suggest that candidate inhibitory factors within the DG, e.g., extracellular matrix molecules, soluble neurotrophic factors, surface molecules on axons, or repulsive molecules on glial cells, do not prevent the ingrowth of crossed entorhinal axons *in vitro*. Whether this is also the case in the intact animal, needs to be addressed in future studies.

If the DG itself allows ingrowth of crossed entorhinal axons, signals along the midline or in the hippocampal commissure could prevent the crossing of entorhinal fibers *in vivo*. However, this does not appear to be likely, since commissural entorhinal axons can cross the midline and reach the contralateral EC. At least in the guinea pig, these fibers also arise from layer II neurons (Christesen and Sorensen, 1989), i.e., the neurons which also give rise to the entorhino-dentate projection. Of note, the crossing fibers arise *via* the ipsilateral angular bundle, cross the midline in the dorsal hippocampal commissure, and reach the contralateral EC *via* the contralateral angular bundle (Christesen and Sorensen, 1989; Adelmann et al., 1996). Thus, commissural entorhinal axons use the same white matter structures as entorhino-dentate axons, suggesting that the angular bundle and the dorsal hippocampal commissure are also permissive, at least in principle, for crossed entorhinal axons.

What could then be a factor limiting the growth of entorhino-dentate fibers *in vivo*? We noted in our material that the crossed projection was generally weaker than the ipsilateral projection and rarely reached the most distal parts of the contralateral molecular layer. These distal parts of the molecular layer were usually filled by a dense plexus of the ipsilateral projection. This situation appears to be somewhat reminiscent of the situation in the rat *in vivo*, in which crossed entorhino-dentate axons are found, albeit only in small numbers and only in the most septal part of the DG, i.e., the part that is first encountered by the contralateral fibers entering the DG *via* the dorsal hippocampal commissure (Steward et al., 1974; Zimmer and Hjorth-Simonsen, 1975). A parsimonious explanation of our observations is that competition between the ipsi- and the crossed entorhinal axons could play a role, as has been shown for other fiber systems in the brain (e.g., Simpson et al., 2009; Ben Fredj et al., 2010): since the ipsilateral axons arrive earlier in the DG, these axons could form synapses first. The later arriving contralateral axons would have to compete with the ipsilateral axons for available synapses. Since the two fiber systems are anatomically and functionally homologous, such a competition situation would be quite physiological and could easily explain a weaker and more limited crossed projection, which expands once the ipsilateral projection is removed (Steward et al., 1974; Zimmer and Hjorth-Simonsen, 1975). Further *in vitro* and *in vivo* experiments are needed to shed light on this question.

What could be the use of such an organotypic system? In recent years, specific contributions of the MEC and LEC to information processing in hippocampal formation have been recognized (Nilssen et al., 2019; Lee et al., 2020). It is now clear, that there are considerable differences between the two cortical regions at the developmental and genetic level and that, nevertheless, the two areas need to interact for normal hippocampal function (Nilssen et al., 2019). The picture that has recently emerged is that the MEC is important for spatial navigation, whereas the LEC relays olfactory information and, possibly, information related to episodic memory and/or object location (reviewed in Nilssen et al., 2019). Although it will require major technological advances before meaningful information can be played into culture systems, such as the one used here, the effects of MEC and LEC inputs onto granule cells can be studied using our preparations. For example, induction of LTP at MEC-granule cell synapses in the middle molecular layer is accompanied by LTD at LEC-granule cell synapses (Abraham et al., 2006, 2007; Jedlicka et al., 2015). These heterosynaptic interactions are mirrored at the structural level: whereas potentiated granule cell spines in the middle molecular layer increase in size, spines of the same cell located in the non-stimulated outer molecular layer decrease in size (Jungenitz et al., 2018). So far, such interactions were studied *in vivo*, requiring considerable effort. In comparison, in organotypic EC-DG double cultures, both the ipsi- and the contralateral EC are readily accessible, and, very importantly, cells of origin and target cells are located within the same plane. The use of optogenetics in combination with LED-assemblies (“cell-culture discos”; Grossman et al., 2010; Wefelmeyer et al., 2015) will open up further possibilities. These tools could be used to play in complex network patterns of activity, which have been previously recorded under physiological conditions, e.g., from freely moving animals. Or they could make it possible to chronically stimulate the perforant path in the hope to create an *in vitro* model of experimental temporal lobe epilepsy (Sloviter, 1987; Kienzler et al., 2006, 2009).

## Data Availability Statement

The original contributions presented in the study are included in the article, further inquiries can be directed to the corresponding author.

## Ethics Statement

All animal experiments were performed in accordance with the German animal welfare law and had been declared to the Animal Welfare Officer of the Faculty (Wa-2014-35).

## Author Contributions

LH-E, MP, CS, NZ, and KY acquired and analyzed the data. ML and AV provided methods. TD conceived and TD, AV, and AD supervised the study. LH-E, MP, AD, and TD wrote the manuscript. All authors were involved in critically revising the manuscript and approved the submitted version.

## Conflict of Interest

The authors declare that the research was conducted in the absence of any commercial or financial relationships that could be construed as a potential conflict of interest.

## Footnotes

^1^http://n2t.net/addgene:26969

## References

[B1] Abraham W. C. Logan B. Wolff A. Benuskova L. (2007). “Heterosynaptic” LTD in the dentate gyrus of anesthetized rat requires homosynaptic activity. J. Neurophysiol. 98, 1048–1051. 10.1152/jn.00250.2007 17537906

[B2] Abraham W. C. Mason-Parker S. E. Irvine G. I. Logan B. Gill A. I. (2006). Induction and activity-dependent reversal of persistent LTP and LTD in lateral perforant path synapses *in vivo*. Neurobiol. Learn. Mem. 86, 82–90. 10.1016/j.nlm.2005.12.007 16458543

[B3] Adelmann G. Deller T. Frotscher M. (1996). Organization of identified fiber tracts in the rat fimbria-fornix: an anterograde tracing and electron microscopic study. Anat. Embryol. 193, 481–493. 10.1007/BF00185879 8729966

[B4] Ben Fredj N. Hammond S. Otsuna H. Chien C. B. Burrone J. Meyer M. P. (2010). Synaptic activity and activity-dependent competition regulates axon arbor maturation, growth arrest, and territory in the retinotectal projection. J. Neurosci. 30, 10939–10951. 10.1523/JNEUROSCI.1556-10.2010 20702722 PMC6634700

[B6] Ceranik K. Deng J. Heimrich B. Lübke J. Zheng S. Förster E.. (1999). Hippocampal Cajal-Retzius cells project to the entorhinal cortex: retrograde tracing and intracellular labelling studies. Eur. J. Neurosci. 11, 4278–4290. 10.1046/j.1460-9568.1999.00860.x 10594654

[B7] Christesen H. B. Sorensen K. E. (1989). The topographical and laminar organization of a commissural-associational entorhino-entorhinal projection in the guinea pig. Brain Res. 505, 75–82. 10.1016/0006-8993(89)90117-0 2611680

[B8] Cotman C. W. Gentry C. Steward O. (1977). Synaptic replacement in the dentate gyrus after unilateral entorhinal lesion: electron microscopic analysis of the extent of replacement of synapses by the remaining entorhinal cortex. J. Neurocytol. 6, 455–464. 10.1007/BF01178228 894334

[B9] del Rio J. A. Heimrich B. Borrell V. Förster E. Drakew A. Alcantara S.. (1997). A role for Cajal-Retzius cells and reelin in the development of hippocampal connections. Nature 385, 70–74. 10.1038/385070a0 8985248

[B10] Del Turco D. Deller T. (2007). Organotypic entorhino-hippocampal slice cultures—a tool to study the molecular and cellular regulation of axonal regeneration and collateral sprouting *in vitro*. Methods Mol. Biol. 399, 55–66. 10.1007/978-1-59745-504-6_5 18309925

[B11] Del Turco D. Paul M. H. Beeg Moreno V. J. Hildebrandt-Einfeldt L. Deller T. (2019). Re-innervation of the denervated dentate gyrus by sprouting associational and commissural mossy cell axons in organotypic tissue cultures of entorhinal cortex and hippocampus. Front. Mol. Neurosci. 12:270. 10.3389/fnmol.2019.00270 31798410 PMC6861856

[B12] Deller T. Del Turco D. Rappert A. Bechmann I. (2007). Structural reorganization of the dentate gyrus following entorhinal denervation: species differences between rat and mouse. Prog. Brain Res. 163, 501–528. 10.1016/S0079-6123(07)63027-1 17765735

[B13] Deller T. Frotscher M. Nitsch R. (1996). Sprouting of crossed entorhinodentate fibers after a unilateral entorhinal lesion: anterograde tracing of fiber reorganization with *Phaseolus vulgaris*-leucoagglutinin (PHAL). J. Comp. Neurol. 365, 42–55. 10.1002/(SICI)1096-9861(19960129)365:1<42::AID-CNE4>3.0.CO;2-J 8821440

[B14] Deller T. Haas C. A. Frotscher M. (2000). Reorganization of the rat fascia dentata after a unilateral entorhinal cortex lesion. Role of the extracellular matrix. Ann. N Y Acad. Sci. 911, 207–220. 10.1111/j.1749-6632.2000.tb06728.x 10911876

[B15] Deller T. Leranth C. (1990). Synaptic connections of neuropeptide Y (NPY) immunoreactive neurons in the hilar area of the rat hippocampus. J. Comp. Neurol. 300, 433–447. 10.1002/cne.903000312 2266195

[B16] Förster E. Zhao S. Frotscher M. (2006). Laminating the hippocampus. Nat. Rev. Neurosci. 7, 259–267. 10.1038/nrn1882 16543914

[B17] Freund T. F. Buzsàki G. (1996). Interneurons of the hippocampus. Hippocampus 6, 345–470. 10.1002/(SICI)1098-1063(1996)6:4<347::AID-HIPO1>3.0.CO;2-I 8915675

[B18] Frotscher M. Heimrich B. (1993). Formation of layer-specific fiber projections to the hippocampus *in vitro*. Proc. Natl. Acad. Sci. U S A 90, 10400–10403. 10.1073/pnas.90.21.10400 8234306 PMC47782

[B19] Frotscher M. Heimrich B. Deller T. Nitsch R. (1995). Understanding the cortex through the hippocampus: lamina-specific connections of the rat hippocampal neurons. J. Anat. 187, 539–545. 8586554 PMC1167458

[B20] Grimm D. (2002). Production methods for gene transfer vectors based on adeno-associated virus serotypes. Methods 28, 146–157. 10.1016/s1046-2023(02)00219-0 12413413

[B21] Grimm D. Kern A. Rittner K. Kleinschmidt J. A. (1998). Novel tools for production and purification of recombinant adenoassociated virus vectors. Hum. Gene. Ther. 9, 2745–2760. 10.1089/hum.1998.9.18-2745 9874273

[B22] Grossman N. Poher V. Grubb M. S. Kennedy G. T. Nikolic K. McGovern B.. (2010). Multi-site optical excitation using ChR2 and micro-LED array. J. Neural Eng. 7:16004. 10.1088/1741-2560/7/1/016004 20075504

[B23] Hjorth-Simonsen A. Zimmer J. (1975). Crossed pathways from the entorhinal area to the fascia dentata: I. Normal in rabbits. J. Comp. Neurol. 161, 57–70. 10.1002/cne.901610106 1133227

[B24] Jedlicka P. Benuskova L. Abraham W. C. (2015). A voltage-based STDP rule combined with fast BCM-like metaplasticity accounts for LTP and concurrent “heterosynaptic” LTD in the dentate gyrus *in vivo*. PLoS Comput. Biol. 11:e1004588 10.1371/journal.pcbi.1004588 26544038 PMC4636250

[B25] Jones R. S. G. (1993). Entorhinal-hippocampal connections: a speculative view of their function. Trends Neurosci. 16, 58–64. 10.1016/0166-2236(93)90018-h 7680501

[B26] Jungenitz T. Beining M. Radic T. Deller T. Cuntz H. Jedlicka P.. (2018). Structural homo- and heterosynaptic plasticity in mature and adult newborn rat hippocampal granule cells. Proc. Natl. Acad. Sci. U S A 115, E4670–E4679. 10.1073/pnas.1801889115 29712871 PMC5960324

[B27] Kienzler F. Jedlicka P. Vuksic M. Deller T. Schwarzacher S. W. (2006). Excitotoxic hippocampal neuron loss following sustained electrical stimulation of the perforant pathway in the mouse. Brain Res. 1085, 195–198. 10.1016/j.brainres.2006.02.055 16580650

[B28] Kienzler F. Norwood B. A. Sloviter R. S. (2009). Hippocampal injury, atrophy, synaptic reorganization, and epileptogenesis after perforant pathway stimulation-induced status epilepticus in the mouse. J. Comp. Neurol. 515, 181–196. 10.1002/cne.22059 19412934 PMC2705826

[B29] Kolodkin A. L. (1996). Growth cones and the cues that repel them. Trends Neurosci. 19, 507–513. 10.1016/S0166-2236(96)10057-6 8931278

[B31] Lee J. H. Durand R. Gradinaru V. Zhang F. Goshen I. Kim D. S.. (2010). Global and local fMRI signals driven by neurons defined optogenetically by type and wiring. Nature 465, 788–792. 10.1038/nature09108 20473285 PMC3177305

[B30] Lee H. GoodSmith D. Knierim J. J. (2020). Parallel processing streams in the hippocampus. Curr. Opin. Neurobiol. 64, 127–134. 10.1016/j.conb.2020.03.004 32502734 PMC8136469

[B32] Leranth C. Malcolm A. J. Frotscher M. (1990). Afferent and efferent synaptic connections of somatostatin-immunoreactive neurons in the rat fascia dentata. J. Comp. Neurol. 295, 111–122. 10.1002/cne.902950110 1971287

[B33] Nilssen E. S. Doan T. P. Nigro M. J. Ohara S. Witter M. P. (2019). Neurons and networks in the entorhinal cortex: a reappraisal of the lateral and medial entorhinal subdivisions mediating parallel cortical pathways. Hippocampus 29, 1238–1254. 10.1002/hipo.23145 31408260

[B34] Reeves T. M. Steward O. (1986). Emergence of the capacity for LTP during reinnervation of the dentate gyrus: evidence that abnormally shaped spines can mediate LTP. Exp. Brain Res. 65, 167–175. 10.1007/BF00243839 3803502

[B35] Schindelin J. Arganda-Carreras I. Frise E. Kaynig V. Longair M. Pietzsch T.. (2012). Fiji: an open-source platform for biological-image analysis. Nat. Methods. 9, 676–682. 10.1038/nmeth.2019 22743772 PMC3855844

[B36] Shevtsova Z. Malik J. M. Michel U. Bahr M. Kugler S. (2005). Promoters and serotypes: targeting of adeno-associated virus vectors for gene transfer in the rat central nervous system *in vitro* and *in vivo*. Exp. Physiol. 90, 53–59. 10.1113/expphysiol.2004.028159 15542619

[B37] Simpson H. D. Mortimer D. Goodhill G. J. (2009). Theoretical models of neural circuit development. Curr. Top. Dev. Biol. 87, 1–51. 10.1016/S0070-2153(09)01201-0 19427515

[B38] Skutella T. Nitsch R. (2001). New molecules for hippocampal development. Trends Neurosci. 24, 107–113. 10.1016/s0166-2236(00)01717-3 11164941

[B39] Sloviter R. S. (1987). Decreased hippocampal inhibition and a selective loss of interneurons in experimental epilepsy. Science. 235, 73–76. 10.1126/science.2879352 2879352

[B40] Steward O. (1976). Reinnervation of dentate gyrus by homologous afferents following entorhinal cortical lesions in adult rats. Science 194, 426–428. 10.1126/science.982024 982024

[B42] Steward O. Cotman C. W. Lynch G. S. (1973). Re-establishment of electrophysiologically functional entorhinal cortical input to the dentate gyrus deafferented by ipsilateral entorhinal lesions: innervation by the contralateral entorhinal cortex. Exp. Brain Res. 18, 396–414. 10.1007/BF00239108 4778785

[B41] Steward O. Cotman C. W. Lynch G. (1974). Growth of a new fiber projection in the brain of adult rats: re-innervation of the dentate gyrus by the contralateral entorhinal cortex following ipsilateral entorhinal lesions. Exp. Brain Res. 20, 45–66. 10.1007/BF00239017 4367724

[B43] Steward O. Vinsant S. L. Davis L. (1988). The process of reinnervation in the dentate gyrus of adult rats: an ultrastructural study of changes in presynaptic terminals as a result of sprouting. J. Comp. Neurol. 267, 203–210. 10.1002/cne.902670205 3343397

[B44] Tamamaki N. (1997). Organization of the entorhinal projection to the rat dentate gyrus revealed by Dil anterograde labeling. Exp. Brain Res. 116, 250–258. 10.1007/pl00005753 9348124

[B45] van Groen T. Kadish I. Wyss J. M. (2002). Species differences in the projections from the entorhinal cortex to the hippocampus. Brain Res. Bull. 57, 553–556. 10.1016/s0361-9230(01)00683-9 11923027

[B46] van Groen T. Miettinen P. Kadish I. (2003). The entorhinal cortex of the mouse: organization of the projection to the hippocampal formation. Hippocampus 13, 133–149. 10.1002/hipo.10037 12625464

[B47] Van Hoesen G. W. Pandya D. N. Butters N. (1972). Cortical afferents to the entorhinal cortex of the Rhesus monkey. Science 175, 1471–1473. 10.1126/science.175.4029.1471 4622430

[B48] Wefelmeyer W. Cattaert D. Burrone J. (2015). Activity-dependent mismatch between axo-axonic synapses and the axon initial segment controls neuronal output. Proc. Natl. Acad. Sci. U S A 112, 9757–9762. 10.1073/pnas.1502902112 26195803 PMC4534224

[B49] Wilson R. C. Steward O. (1978). Polysynaptic activation of the dentate gyrus of the hippocampal formation: an olfactory input *via* the lateral entorhinal cortex. Exp. Brain. Res 33, 523–534. 10.1007/BF00235572 215436

[B50] Zimmer J. Hjorth-Simonsen A. (1975). Crossed pathways from the entorhinal area to the fascia dentata: II. Provokable in rats. J. Comp. Neurol. 161, 71–101. 10.1002/cne.901610107 1133228

